# Potential crosstalk between cofilin-1 and EGFR pathways in cisplatin resistance of non-small-cell lung cancer

**DOI:** 10.18632/oncotarget.3471

**Published:** 2015-02-28

**Authors:** Carolina Beatriz Müller, Marco Antônio De Bastiani, Matheus Becker, Fernanda Stapenhorst França, Mariane Araujo Branco, Mauro Antônio Alves Castro, Fábio Klamt

**Affiliations:** ^1^ Laboratory of Cellular Biochemistry, Department of Biochemistry, Federal University of Rio Grande do Sul (UFRGS), Porto Alegre (RS), Brazil; ^2^ National Institutes for Science & Technology-Translational Medicine (INCT-TM), Porto Alegre (RS), Brazil; ^3^ Programa de Pós-Graduação em Bioinformática, Federal University of Paraná (UFPR), Curitiba (PR), Brazil

**Keywords:** NSCLC, EGFR, cofilin-1, chemotherapy resistance, personalized medicine

## Abstract

Current challenge in oncology is to establish the concept of personalized medicine in clinical practice. In this context, non-small-cell lung cancer (NSCLC) presents clinical, histological and molecular heterogeneity, being one of the most genomically diverse of all cancers. Recent advances added Epidermal Growth Factor Receptor (EGFR) as a predictive biomarker for patients with advanced NSCLC. In tumors with activating EGFR mutations, tyrosine kinase inhibitors (TKI) are indicated as first-line treatment, although restricted to a very small target population. In this context, cofilin-1 (a cytosolic protein involved with actin dynamics) has been widely studied as a biomarker of an aggressive phenotype in tumors, and overexpression of cofilin-1 is associated with cisplatin resistance and poor prognosis in NSCLC. Here, we gather information about the predictive potential of cofilin-1 and reviewed the crosstalk between cofilin-1/EGFR pathways. We aimed to highlight new perspectives of how these interactions might affect cisplatin resistance in NSCLC. We propose that cofilin-1 quantification in clinical samples in combination with presence/absence of EGFR mutation could be used to select patients that would benefit from TKI's treatment. This information is of paramount importance and could result in a possibility of guiding more effective treatments to NSCLC patients.

## INTRODUCTION

The current challenge in oncology is to establish the concept of personalized medicine in clinical practice [1]. Classification into subpopulations differed by their susceptibility to a particular disease and response to a specific treatment allows therapeutic intervention to be focused on patients who will greatly benefit from it, sparing those who will not [2].

For cancer therapeutics, the use of specific characteristics of mutational status and deregulated pathways of tumor itself might help to prevent, diagnose and treat the disease [3]. The central hypothesis is that treatment decisions based on tumor genotype and genomic profile, correlated with clinical factors, would improve clinical outcomes, as measured by response rate, survival and safety [4]. Furthermore, to guarantee that patients can access personalized medicine, a new paradigm has evolved, the “P4” (standing for *p*redictive, *p*reventive, *p*ersonalized and *p*articipatory medicine), based on scientific, organizational and wellness strategies. Thus, to achieve that, oncology will have to move from a reactive to a proactive discipline [5].

This approach has good application to heterogeneous disorders, such as lung cancer whose development and manifestation vary greatly from patient to patient. Lung cancer is a disease with clinical, histological and molecular heterogeneity, remaining one of the leading causes of cancer mortality worldwide [6]. The lethality of this disease can be attributed to late diagnosis (hindering the possibility for surgical treatment), resistance to chemotherapy treatments and emerging of complications in advanced stages [7]. Additionally, traditional lung cancer chemotherapy is not curative and provides limited benefits, with average survival of less than one year. Nevertheless, we faced a decade of significant advances in the identification of key driver events in lung carcinogenesis and target lung cancer therapies [8]. The most prevalent type of lung cancer is non-small-cell lung cancer (NSCLC). It is also described as one of the most genomically diverse of all cancers [9]. This feature imposes a great challenge for prevention and treatment strategies, but at the same time provides a number of opportunities for intervention by ungrouping NSCLC into a variety of molecularly defined subsets [4, 6]. In view of such challenges, finding biomarkers that could overcome these obstacles and group patients according to optimal responsiveness and efficacy, would lead to a better treatment and management.

Recent advances added EGFR (Epidermal Growth Factor Receptor) and ALK (Anaplastic Lymphoma Kinase) as biomarkers that should be tested for in patients with advanced lung cancer. For tumors with activating EGFR mutations (e.g.: L858R and E746-A750del), EGFR tyrosine kinase inhibitors (EGFR-TKI) (such as gefitinib, erlotinib, and afatinib) are indicated as first-line treatment [10]. Although this treatment is already in clinical practice, there is still controversy about its effect on patients overall survival (OS); in addition, it seems to be very restricted to a target population composed primarily of non-smoking women with adenocarcinoma [11].

In this context, cofilin-1 – a small protein of 18 kDa – has been widely studied as a biomarker of a more aggressive phenotype of different types of cancer such as breast, gastrointestinal and NSCLC [12-14]. The comprehension of its association with EGFR and relation with conventional alkylating agent-based therapy resistance, could help to discriminate and increase the suitable population to TKI's treatment. Here, we gather information about cofilin-1 therapeutic prediction potential and review the crosstalk between cofilin-1 and EGFR pathways, highlighting new perspectives of how these interactions might affect cisplatin resistance in NSCLC.

### Cofilin-1 and its predictive role in cancer chemotherapy

Cofilin-1 (*CFL1*; non-muscle isoform; Gene ID: 1072) is a conserved and ubiquous protein in mammals, classically involved with actin polymerization/depolymerization dynamics [15]. In the last decade, however, new and unexpected roles of this protein have been described in other pathological and physiological cellular situations, such as apoptosis induced by oxidants [16] and intracellular rods formation in neurodegenerative diseases [17-19].

Over the last 20 years, several studies have pointed cofilin-1 as an important protein in aggressive cancer cell behavior, due to its involvement in the coordination of tumor cell migration and invasion [12, 20-24]. There are four important mechanisms that regulate the activation status of cofilin-1: (1) its dephosphorylation at Ser3; (2) its release from phosphatidylinositol-4,5bisphosphate (PtdIns(4,5)P2); (3) its release from cortactin; and (4) regulation by oxidation/reduction of one of its four cysteins residues [16]. Dephosphorylation of cofilin-1 at Ser3 was the first activation mechanism to be well characterized. Slingshot (SSH) was shown to be a major phosphatase responsible for dephosphorylating cofilin-1 at Ser3, and chronophin (CIN) was recently identified as a cofilin-1 specific phosphatase. In addition, the serine-phosphatases PP1 and PP2A can also dephosphorylate cofilin-1 at Ser3. On the other hand, LIMK1 and LIMK2 as well as TES kinase 1 (TESK1) and TESK2 phosphorylate cofilin-1 at Ser3 *in vivo*. LIMK1/2 are the most well studied kinases and have been proposed to be the dominant kinase in the regulation of actin dynamics by mediating cofilin-1 inactivation. Cofilin-1 can still be inactivated by its interaction with PtdIns(4,5)P2 at the plasma membrane. This follows a general mechanism whereby membrane lipids have been shown to bind various actin regulatory proteins. In migrating cells, the hydrolysis of PtdIns(4,5)P2 can release cofilin-1 from its inhibitory interaction with the membrane lipids, resulting in the local activation of F-actin filament severing, protrusion and cell polarity. Finally, the binding of cofilin-1 to the actin regulatory protein cortactin also negatively regulates cofilin-1 activity, and this mechanism seems to be specific to invadopodia formation [12, 25, 26]. Deregulations of such pathways, favoring tumorigenesis, have been described in some extension for different types of carcinomas, like breast, oral, ovarian, prostate, melanoma and gastrointestinal cancer, indicating a strong prognostic correlation [12, 13, 27-31].

**Figure 1:**
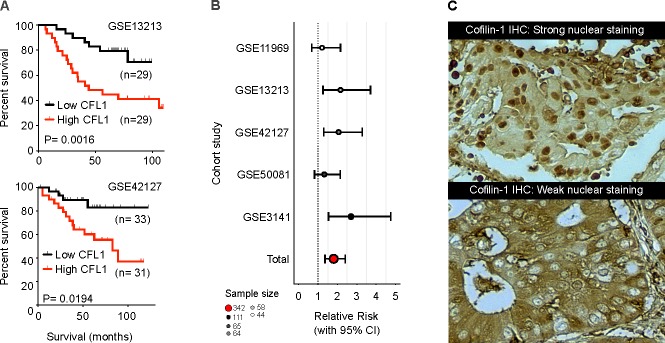
Meta-analysis results of cofilin-1 prognostic potential (A) Kaplan-Meier mortality curves indicating *CFL1* strength in predicting patient survival. (B) Forest plot of five different studies showing relative risk of death in high expressing *CFL1* mRNA patients. Microarray data were obtained from Gene Expression Omnibus (GEO) online repository (http://www.ncbi.nlm.nih.gov/geo/). (C) Immunohistochemistry for cofilin-1 in two different NSCLC slides, presenting presence/absence of nuclear staining.

Regarding NSCLC, a series of correlational studies using meta-analysis of microarray data showed that mRNA level of *CFL1* in NSCLC can discriminate between good and bad prognosis, in which tumors with high expression of *CFL1* are associated with low overall survival (OS) [14, 32]. This microarray data was validated in a retrospective NSCLC cohort by a semi-quantitative immunohistochemistry method [33]. Meta-analysis of other independent cohorts microarray data also corroborates that cofilin-1 has a prognostic capability, indicating that patients with higher levels of this protein are more likely to be at the poorer outcome group (Figure 1). In these works, however, the relation of cofilin-1′s expression with a more aggressive phenotype of tumors was attributed to its classical activity upon actin cytoskeleton modulation, related to improved migration and invasion capacity in cancer cells, as reviewed recently [26]. Moreover, NSCLC cell lines with high cofilin-1 immunocontent have high invasive potential and were found to be resistant to cisplatin and carboplatin treatment (compounds that are gold-standard drugs used in NSCLC patient management), indicating that cofilin-1 might also present a predictive aspect to be explored [14].

Hints of a possible role of cofilin-1 in the cellular resistance against alkylating agents have been described in cisplatin/carboplatin resistant ovarian cell lines almost 10 years ago [34]. Regarding NSCLC, available data from pre-clinical studies point to the same direction [14, 35, 36]. Analysis of microarray data in a drug screening cell panel (NCI60 cell panel) of 118 chemotherapeutic compounds showed that *CFL1* mRNA level is correlated with resistance against 21 of 30 alkylating agents (such as cisplatin and carboplatin) tested [14]. High levels of cofilin-1 were found in cisplatin-resistant A549 NCSLC cells and A549 cells transiently overexpressing *CFL1* plasmid present an increased in GI_50_ value for cisplatin [36]. Wei and collaborators also found high levels of cofilin-1 in cisplatin-resistant NSCLC cell lines using proteomics studies [35]. These studies support the idea that high level of cofilin-1 correlates with cisplatin resistance.

Several mechanisms account for the cisplatin-resistant phenotype of tumor cells. Most described are drug reduced uptake/increased efflux (mediated mainly by the plasma membrane copper transporter CTR1, copper-extruding P-type ATPases ATP7A/ATP7B, and members of the ABC family of transporters MRP and MDR), increased inactivation (by GSH/γ-GCS/GST and metallothioneins), and increased repair capacity of DNA lesions (mediated by members of the nucleotide excision repair pathway such as ERCC1 or by the machinery for homologous recombination BRCA1/BRCA2) [37]. Cisplatin cytotoxic is described by its interaction with nucleophilic sites in N7 position of purines in DNA, forming DNA-protein interactions, inter and intra-strands crosslinks and DNA adducts [38], which are the main lesions responsible for cell death [39]. More than 90% of cisplatin-DNA adducts result in crosslinks 1.2 d (GpG) intra-strands, which modifies the three dimensional structure of the DNA molecule, enabling this site for several proteins recognition. These proteins include damage recognition components of the mismatch repair (MMR) complex, such as group 1 and 2 proteins of non-histone high mobility group of proteins (HMG1 and HMG2), proteins related to nucleotide excision repair (NER), among others [38, 40]. In this scenario, the precise mechanism that leads to cisplatin resistance is not well established. Cofilin-1 presents a nuclear localization signal in its primary structure and can translocate into the nucleus under specific chemical or physical stimuli (Figure 1) [41, 42]. These information hints the possibility that cofilin-1 could have a nuclear role in supporting the DNA repair system.

Although these data could potentially impact an appropriate treatment prediction, many questions related to these events remain to be answered. A *sine qua non* condition to use this information in patient benefit is to visualize cofilin-1 pathway interactions and how this might affect cellular resistance machinery.

### EGFR: a biological marker in clinical practice

The EGF receptor (EGFR) belongs to the ErbB family of receptor tyrosine kinase (RTK) greatly known for its involvement with pro-tumorigenic pathways [43]. EGFR, or HER1, is one of a family of epidermal growth factor (EGF) receptors that also includes ErbB2/HER-2, ErbB3/HER-3, and ErbB4/HER-4. Binding of its ligands result in conformational change of EGFR, homodimerization or heterodimerization with other members of the receptor family, and autophosphorylation of the cytoplasmic tyrosine kinase domain. EGFR signaling network has an interactive nature, being one of the most deregulated molecular pathways found in human cancer. The major pathways downstream EGFR activation are Ras/Raf/MEK, PI3K/AKT/mTOR, JAK2/STAT3 and PLC-gamma/PKC [43-45]. All these pathways are important for tumor growth, progression and survival.

Besides that, EGFR at different subcellular location has different functions and overlapping signals [45]. Therefore, various strategies of targeting EGFR or its family members have been developed and are in different phases of clinical trials [46]. However, feedback and crosstalk circuits between signaling pathways could limit the selection of one driven gene mutation for treatment with a matching drug. This underlines the difficulty of using a single marker to predict patient susceptibility to a particular disease and response to a specific treatment. Another important factor of tumor aggressiveness is the potential cell migration and ability to leave primary tumor sites. In this aspect, EGF has been shown to be an important chemotactic molecule both in physiological and in pathological situations [47]. In fact, in MDA-MB-231 breast cancer cells, PI3K and PLC-gamma pathways indeed promote migration [48]. Thus, research to identify active pathways downstream EGFR activation could lead the rationale for the development of multidrug combination therapies striking several critical points important to tumor development [49].

### Cytosolic and nuclear crosstalks between Cofilin-1 and EGFR pathways

**Figure 2:**
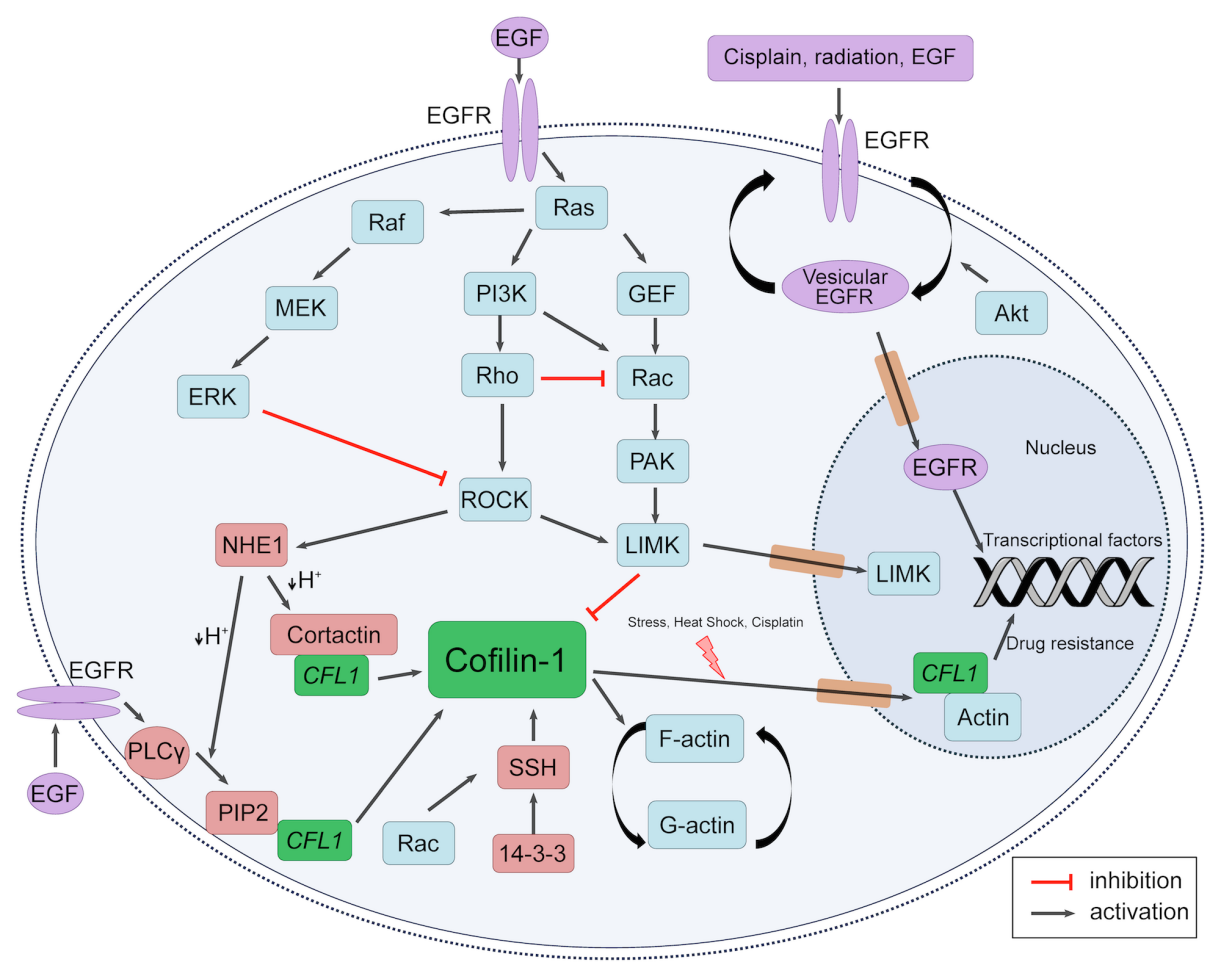
EGFR and Cofilin-1 cytosolic and nuclear crosstalk Schematic representation of EGFR and cofilin-1 pathways intersections. Different local stimuli may result in cofilin-1 modulation through EGFR activation. Downstream EGFR pathways may activate cofilin-1 through dephosphorylation by SSH1 and release of cortactin and PIP_2_ bounds by intracellular pH alteration; also, it may result in cofilin-1 inactivation by LIMK activity. Cofilin-1 and EGFR may also translocate into nucleus in response to external stimuli, indicating a possibility of related mechanisms of drug resistance.

There is an intense crosstalk between EGFR and cofilin-1 pathways, as summarized in Figure 2. Indeed, EGFR downstream routes indirectly regulate all of the described cofilin-1 activation/inactivation mechanisms. Cofilin-1′s major kinase, LIMK1, is modulated via EGFR-PI3K route. PI3K activates small Rho GTPases such as Rac and CDC42, which mediate activation of p21-activated kinase 1 (PAK1) and Rho-dependent protein kinase 1 (ROCK1). Afterwards, these kinases phosphorylate, and activate LIMK [50-53]. On the other hand, cofilin-1 dephosphorylation by SSH1 may also be modulated downstream EGFR [54]. As Kligys and collaborators have demonstrated, SSH1 activation occurs via Rac1 in keratinocytes [55]. Moreover, it is well established that EGFR signaling activates Rac1 [56]. Therefore, EGFR pathway can modulate the phosphorylation (and so the activation) state of cofilin-1.

Another intersection between EGFR and cofilin-1 pathways is via PLC gamma activation followed by PtdIns(4,5)P2 hydrolysis, an important mechanism of local cofilin-1 mobilization [25, 57]. Lastly, *tyr* phosphorylation of cortactin by Arg kinase, which is activated downstream of EGFR, regulates the interaction between the Na^+^-H^+^ exchanger 1 (NHE1) and cortactin. NHE1 increases the intracellular pH, which induces the release of cortactin-bound cofilin-1 [58, 59]. Therefore, EGFR pathway plays a pivotal role over cofilin-1 activity states in response to different cellular stimuli, leading to several ways to modulate cell adaptation either in pathological as well as physiological situations.

Nuclear localization of EGFR was first observed more than two decades ago in hepatocytes [60]. Only recently, however, the nuclear translocation of this protein was shown to be induced by several stimuli, such as EGF, ionizing radiation and cisplatin treatment [61]. Activation of EGFR results in its endocytosis and interaction with importin β1 via its tripartite nuclear localization sequence [62]. Moreover, EGFR undergoes to nucleus via a retrograde trafficking from Golgi apparatus to ER. Once embedded into the ER membrane, EGFR and importin β1 interface with nucleoporins in the nuclear pore complex (NPC) to shuttle EGFR from the outer nuclear membrane (ONM) to the inner nuclear membrane (INM) [63, 64]. Once EGFR is inside the nucleus, it may display four major functions: *i*) promote gene regulation (an independent kinase activity of EGFR), acting as a co-factor and increasing expression of target genes, like iNOS, COX-2, c-Myc, cyclins and others, contributing to several malignant phenotypes of human cancers; *ii*) phosphorylates proliferating cell nuclear antigen (PCNA), promoting its stability and contributing to cell proliferation and DNA repair (an activity dependent of its kinase activity); *iii*) interacts with DNA-dependent protein kinase (DNA-PK) and enhances the DNA repair machinery; *iv*) co-localizes with γH_2_AX complex, enabling chromatin relaxation for DNA repair process [65, 66]. Therefore, it is not surprising that a growing body of evidence has demonstrated a strong association between nuclear EGFR and resistance to chemotherapy/radiotherapy in tumors.

It has been reported that cisplatin stimuli can induce EGFR activity and downstream events and this process is ligand-independent [67]. Regarding cisplatin resistance, murine NIH-3T3 fibroblasts cells treated with cisplatin had an increasing in nuclear EGFR associated with DNA-PKs, which contributed to cisplatin resistance [61]. This involvement of nuclear EGFR and DNA-PK enhancing DNA repair and cisplatin resistance was also demonstrated in human tumor cell lines [68]. Moreover, nuclear EGFR was correlated with shorter progression-free survival in early NSCLC stage [69]. This association with poor prognosis is in accordance with the fact that nuclear EGFR activity was related to tumor radio and chemoresistance. However, it is not yet clear how nuclear EGFR affects TKI and antibodies target therapies.

On the other hand, nuclear translocation of cofilin-1 was first described in 1987 by Nishida and collaborators in mouse fibroblast cell line C3H-2K stimulationed with 10% of dimethyl sulfoxide (DMSO) or heat shock at 42-43°C for 60 minutes [70]. Afterward, studies have shown that cofilin-1 nuclear translocation upon such stimuli requires dephosphorylation at serine-3 domain to expose its nuclear localization signal (NLS). Moreover, cofilin-1 seems to play an important role in cellular stress contexts by leading monomeric actin (G-actin) inside the nucleus, since G-actin does not have NLS [41, 71-73]. For example, Sotiropoulos and colleagues showed that monomeric actin is able to inhibit SRF (serum response factor)-dependent gene transcription activation inside the nucleus [74]. However, cofilin-1 appears to have functions besides actin translocation when inside the nucleus. Indeed, studies have pointed a direct role of cofilin-1 in modulation of transcription independently of actin [73, 75]. Additionally, the regulation of cofilin-1 inside the nucleus may also contribute to phenotype changes, since nuclear LIMK enhances human breast cancer progression [76]. Hence, the roles cofilin-1 may play inside the nucleus are still a prospect for further studies. Likewise, there are no studies trying to associate nuclear cofilin-1 with patient's outcome/prognosis in lung cancer.

## CONCLUSIONS AND FUTURE DIRECTIONS

Considering the information gathered here, it seems clear that cofilin-1 regulation and functions are closely related to EGFR activity. However, some evidences allow the assumption of a greater extent of these interactions. EGFR functions inside the nucleus have been subject of intense study, leading to many possible roles of its translocation upon several stimuli [77]. As presented in Figure 2, cisplatin is one of these stimuli, which may lead to nuclear EGFR translocation in tumor cells and resistance to treatment, as result of an enhanced DNA repair [62]. In this same scenario, we have described a positive correlation between cofilin-1 expression and cisplatin resistance in NSCLC cell lines [14, 36]. Considering these facts, could cofilin-1 be affecting EGFR translocation to the nucleus? Indeed, cofilin-1 signaling plays a pivotal role in the regulation of efficient EGFR vesicular trafficking in invasive tumor cell [78, 79].

Since cofilin-1 has a nuclear location signal (NLS) and may enter into nucleus, as presented in Figure 1C, would its activity be restricted to EGFR vesicular trafficking? Could nuclear cofilin-1 also play a direct role in the resistance mechanism to platinum compound? Dopie and colleagues have shown that actin constantly shuttles between cytoplasm and nucleus and they assign to cofilin-1 the role of regulating this continuous *steady-state* actin flow [73]. Based on this, cofilin-1 could be necessary to maintain a *pool* of actin inside the nucleus thus maintaining a “nuclearskeleton” of actin. This could contribute to the transcriptional action of EGFR within the nucleus. On the other hand, cofilin-1, as well as EGFR, can act directly on transcription. According to Obrdlik and Percipalle, cofilin-1 is a key regulator of pol II transcription and its interaction with actin would facilitate the association of transcription machinery with actively transcribed genes [75].

Therefore, seems that cofilin-1 and EGFR pathways are closely related in driving the resistance machinery to cisplatin. Further studies that could evaluate co-localization and activity of cofilin-1 and EGFR in cancer cells would help to elucidate how exactly they are working together towards resistance behavior against cisplatin treatment. Given that increased expression of cofilin-1 is directly related to cisplatin resistance, we propose that its quantification could be used in association with presence/absence of EGFR mutation to guide which patients would benefit better from TKI's treatment. Moreover, studies associating both variables with patient's outcome could better elucidate this relationship. This information is of paramount importance and may, ultimately, result in a possibility of guiding more effective treatments to NSCLC patients, potentially expanding the target population.
